# Human-Induced Trophic Cascades along the Fecal Detritus Pathway

**DOI:** 10.1371/journal.pone.0075819

**Published:** 2013-10-16

**Authors:** Elizabeth Nichols, María Uriarte, Carlos A. Peres, Julio Louzada, Rodrigo Fagundes Braga, Gustavo Schiffler, Whaldener Endo, Sacha H. Spector

**Affiliations:** 1 Department of Ecology, Evolution and Environmental Biology, Columbia University, New York, New York, United States of America; 2 Center for Biodiversity and Conservation, American Museum of Natural History, New York, New York, United States of America; 3 School of Environmental Sciences, University of East Anglia, Norwich, Norfolk, United Kingdom; 4 Departamento de Biologia, Universidade Federal de Lavras, Lavras, Minas Gerais, Brazil; 5 Lancaster Environment Centre, Lancaster University, Lancaster, Lancashire, United Kingdom; 6 Departamento de Biologia, Universidade Estadual de Campinas, São Paulo, Brazil; 7 Department of Ecology and Natural Resource Management, Norwegian University of Life Sciences, As, Norway; 8 Department of Conservation Science, Scenic Hudson, Poughkeepsie, New York, United States of America; University of Toronto, Canada

## Abstract

Human presence and activity in tropical forest is thought to exert top-down regulation over the various ‘green-world’ pathways of plant-based foodwebs. However, these effects have never been explored for the ‘brown-world’ pathways of fecal-detritus webs. The strong effects of humans on tropical game mammals are likely to indirectly influence fecal detritivores (including Scarabaeine dung beetles), with subsequent indirect impacts on detrivore-mediated and plant-facilitating detrital processes. Across a 380-km gradient of human influence in the western Brazilian Amazon, we conducted the first landscape-level assessment of human-induced cascade effects on the fecal detritus pathway, by coupling data on human impact, game mammal and detritivore community structure, and rate measurements of a key detritus process (i.e. dung beetle-mediated secondary seed dispersal). We found evidence that human impact indirectly influences both the diversity and biomass of fecal detritivores, but not detritivore-mediated processes. Cascade strength varied across detritivore groups defined by species' traits. We found smaller-bodied dung beetles were at higher risk of local decline in areas of human presence, and that body size was a better predictor of cascade structure than fecal resource manipulation strategy. Cascade strength was also stronger in upland, unflooded forests, than in seasonally flooded forests. Our results suggest that the impact of human activity in tropical forest on fecal-detritus food web structure is mediated by both species' traits and habitat type. Further research will be required to determine the conditions under which these cascade effects influence fecal-detritus web function.

## Introduction

Abundant evidence now supports a role for vertebrate regulation of the structure and function of foodwebs [1]–[4]. While the majority of trophic cascade research has been conducted along plant-based pathways [5]–[7], comparatively little is known about cascade dynamics in along detritus pathways [7]. In particular, the fecal detritus pathway makes a major contribution to terrestrial nutrient cycles [8] and is expected to be sensitive to top-down regulation of the availability or diversity of fecal detritus inputs [6], [9]. Top-down regulation of the fecal detritus web may arise from tri-trophic indirect interactions, with implications for plants that are either negative (e.g. via predator-mediated reductions in detritivore densities) [5], or positive (e.g. via predator-mediated changes in detritivore behavior) [10].

Alternatively, predator-mediated reduction in herbivore fecal resource availability may instigate four-level trophic cascades along the fecal detritus web. For example, mammal overhunting in tropical forests is predicted to negatively impact fecal detritivore communities by reducing the diversity and availability of fecal detritus inputs from the game mammals targeted by rural hunters [11], [12]. These indirect impacts should further cascade to influence plant growth and demography by reducing rates of detrivore-mediated, plant-facilitating processes including nutrient cycling and secondary seed dispersal [10], [12], [13]. A range of cascading influences of human activity on tropical forest function has been explored along plant-based pathways [14], [15], but these effects have never been examined along fecal detritus-based pathways. Resolving these uncertainties is critical to a more complete understanding of the ecological impacts of human activity in tropical forests.

**Figure 1:**
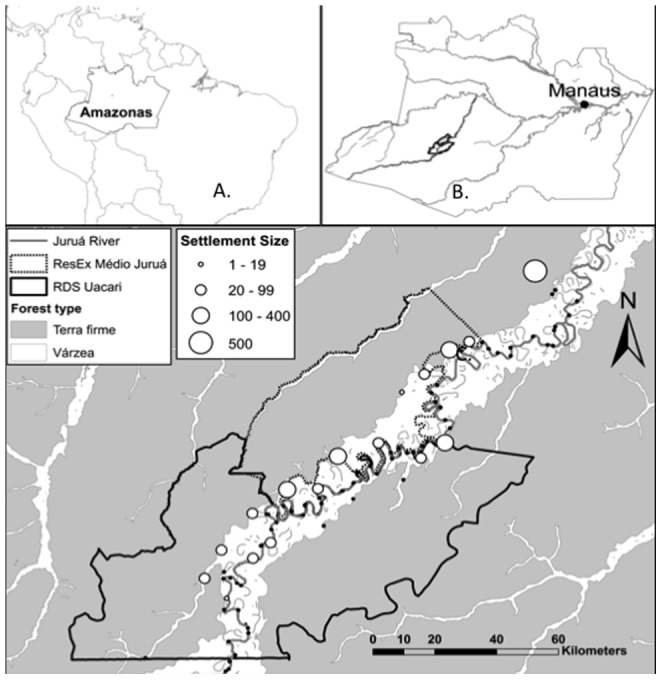
Map of study region. Panels show location of: (top left) the state of Amazonas within Brazil, (top right) the two focal reserves within the state of Amazonas, and (bottom) the distribution of sampled communities (white circles) and settlement size (scaled to size of circle) within and immediately adjacent to the two focal reserves (the Médio Juruá Extractive Reserve and the Uacarí Sustainable Development Reserve). Shaded and white areas indicate *terra firme* and *várzea* forest, respectively.

Along a 380-km gradient of human impact along the Juruá River in the western Brazilian Amazon (Fig. 1), we quantified the influence of human activity on a four-level fecal detritus-based pathway, composed of game mammals, fecal detritivores (i.e. Scarabaeine dung beetles), and fecal detritus process rates (i.e. dung beetle-mediated secondary burial of excreted seeds) [16]. We relied on these data to address two related questions.

First, does human activity in tropical forest influence the community structure of fecal detritivores by decreasing the availability and diversity of mammal fecal inputs? The ability of trophic cascades to substantially alter the biomass or diversity of entire trophic levels (i.e. community-level cascades) remains controversial [17]. Community-wide cascades are considered unlikely in tropical terrestrial habitats [17], [18], as high spatial heterogeneity and high species and trait diversity [19]–[21] may lead to compensatory responses that mask the detection of community-level change [19]. However, top-down regulation of the fecal detritus web may result in community-level responses by fecal detritivores, given their strong dependence on game mammal feces [11], [12] and the lack of density or biomass compensation in perturbed dung beetle communities, particularly within the Neotropics [33].

Alternatively, the strength of trophic cascades may be driven by variation in values of species' trait important for trophic interaction [22]–[25]. Human activity-mediated reductions in the availability and diversity of game mammal feces are hypothesized to disproportionately penalize larger-bodied species whose reproductive output is most closely associated with the large fecal deposits of large-bodied game vertebrates [12]. Fecal resource decline may also differentially influence dung beetle species with different morphologies associated with feces handling and relocation (nesting). Species with ‘tunneler’ and ‘dweller’ strategies appear to be morphologically equipped to manipulate the soft fecal masses produced by those large-bodied primates that are targeted by rural hunters [26]. In contrast, ‘roller’ species commonly manipulate small, pelleted feces, and may more easily handle the feces of non-hunted, smaller-bodied mammals.

Second, can human impact-mediated trophic cascades influence a key detrital process - the secondary dispersal by dung beetles of seeds excreted in mammal feces? We used a field experiment to quantify the proportion of seeds buried by detrivores across a gradient of human impact. We expected that (i) heavier human impact would be correlated with reduced seed burial rates due to the indirect influence of hunting on community-level dung beetle biomass and diversity, (ii) the negative effects of human impact would be disproportionately strong for the largest sized seeds [27], and that (iii) the biomass of large-bodied beetle species would be most strongly correlated with seed dispersal rates across all seed sizes [36].

To better understand the generality of top-down trophic processes in fecal detritus webs, we explored these questions across two common Amazonian forest types. Upland forests (*terra* firme) represent the dominant forest type across the Amazon, and are associated with heavily leached and oligotrophic soils. Seasonally flooded forests (várzea) along white-water rivers account cover roughly 180,000 km^2^ of the 7 million km^2^ (2.6%) of the Amazon basin [28]. These forests differ dramatically in several factors that may influence cascade strength, including edaphic productivity [29], [30], vertebrate diversity [29], [31] and the seasonality and intensity of human resource use and access [32], including patterns of hunting and fishing [33].

## Methods

### Study area

The study was conducted along the Juruá river in the municipal district of Carauari, state of Amazonas, Brazil. The regional vegetation is classified as lowland tropical forest, encompassing a mosaic of 17.9% of *várzea* forest and 80.6% of *terra firme* forests on higher elevations (Fig. 1; [34]).

Data on mammal and dung beetle communities and seed burial rates were collected across a total of 26 sites located within and immediately adjacent to two multiple-use protected areas: the Médio Juruá Extractive Reserve (ResEx; 253,227 ha) and the Uacari Sustainable Development Reserve (RDS; 632,949 ha). These reserves are contiguous and bisected by the Juruá River, a large white-water tributary of the Amazon (Solimões) River (Fig. 1). The two reserves share near-identical extractive histories and ecological, socioeconomic and cultural contexts [35], [36]. Elevation across the study region ranges from 65 to 170 masl. Meteorological data collected daily at the Bauana Ecological Field Station near the center of the study landscape indicated that the mean annual rainfall in 2008–2009 was 4,154 mm. Rainfall is strongly seasonal, with a rainy season from December–May and a persistent flood pulse from January to June.

Within the reserve system, approximately 4,100 local residents are distributed across 74 variable-sized human settlements. Adjacent to the reserve system lies the municipal town of Carauari and the satellite community of Riozinho, with a total estimated population of 25,200 [37]. Reserve residents variously engage in hunting, fishing and forest extraction as well as small-scale agriculture for both local subsistence and commerce [32]. Subsistence hunting is legal in Brazilian multiple-use protected areas and reserve residents hunt with shotguns to supplement an otherwise fish and manioc-based diet.

### Human impact

Human impact was represented as the size of each permanent human settlement (i.e. number of households) nearest each census transect [41]. This measure of human influence on wildlife acts as a proxy for the diversity of ways in which humans impact multi-species game communities, including population response to current and historical human hunting pressure [38], the inducement of avoidance behaviors in game species exposed to human hunting [23], and the impact of localized land-use change [39]. Both settlement age and size (i.e. number of households or hunters) have been successfully used as proxies for the influence of humans on game mammal populations [38], [40]–[42], and provide an estimate of the magnitude of exploitation pressure that is independent of the status of hunted populations [26], [43], [44], hunting effort (e.g. hours hunting/km^2^) [45], [46], frequency [47] or biomass offtake [48]. While human settlement age is often a stronger predictor of game mammal responses [38] than settlement size [26], the dynamism of human settlement patterns in this study region [36], [49] precluded the accurate use of settlement age as a proxy for human impact. In this study region, settlement size was inversely correlated with straight-line distances to the nearest urban center (Carauari, r_25_ = −0.59, p = 0.001).

### Mammal Surveys

Between January 2008 and December 2010, medium and large-bodied (≥1 kg) terrestrial mammal assemblages were characterized using standardized line-transect surveys across a total of 26 sites (*terra firme* forest N = 15, *várzea* forest N = 11) distributed across the study region [50]. Medium to large-bodied mammals represent the preferred game species among traditional hunters [38] and account for a disproportionate fraction of the total vertebrate biomass in tropical forests [51]. Each transect of 4,500–5,000 m in length (mean length: 4,817±337.3 m, n = 26) was surveyed both in the morning and afternoon, over a period of 4–5 consecutive rainless days every month by a trained field assistant from the nearest village, at a mean velocity of 1.2 km/h [50]. Species identity, group size and location were recorded for each encounter. Data on mammal individuals were pooled across space (i.e. along the transect length) and time (i.e. across all census events, 2008–2010), and divided by the total number of kilometers walked, resulting in estimates of abundance corrected for sampling effort for each species observed. For social species, when the number of individuals in a group encounter could not be estimated in the field, the mean group size for that species from the same season and transect was used.

### Dung Beetle Surveys

Dung beetles (Coleoptera: Scarabaeidae: Scarabaeinae) were sampled using standardized baited pitfall traps (20 cm diameter, 15 cm depth) buried flush with the ground and baited with 20 g of fresh human dung. Human feces are routinely used as standardized collection protocol in Neotropical dung beetle biodiversity studies [52], as they attract species beetles known to use both primate, herbivore and omnivore feces [53], are frequently reported to attract a wider breadth of species and higher community biomass than other fecal bait types [53]–[55], and are consistently availability in remote study regions, permitting a minimum level of methodological consistency between studies.

In each of 15 *terra firme* and 11 *várzea* forest sites, a total of 15 traps were placed every 50 m along linear transects, beginning at the 400 m mark of the same transect used for surveying mammals. These trail segments were those nearest to local communities along each transect, thereby maximizing any spatial effects of human activities on the fecal detritus system. Trapping was conducted twice at each site, coinciding with the late-dry (August–September 2009) and early-wet seasons (December–January 2010). Fewer sites were sampled in the wet season due to accessibility issues (*terra firme* N = 10, *várzea* N = 8). Traps were operated for one 24-h period at each site. Captured specimens were separated to species [56]. Dung beetle body mass is a particularly important trait for understanding response to resource availability [9], habitat change [57] and influence on ecological functions, specifically seed dispersal [58]. Body mass estimates for each species were obtained by weighing between 1 and 30 individuals on a balance accurate to 0·0001 g after drying in a constant-temperature oven at 60°C for one week. Nesting strategy information was obtained from the literature and corroborated by experts [57]. Three principal nesting strategies are recognized: *paracoprid* (i.e. tunneler) species locate their nests underneath the fecal deposit; *telocoprid* (i.e. roller) species locate their nests at great horizontal distances from the fecal deposit; and *endocoprid* (i.e. dweller) species nest directly within fecal deposits [59].

### Seed Burial Rates

We set up a seed burial experiment the day before dung beetles were sampled. The sampling protocol consisted of establishing four circular, 1 m diameter mesocosm plots, spaced 100 m apart, and located within the first 400 m of transects used to survey mammal and beetles. Due to logistical constraints, we only measured seed burial within *terra firme* forest (n = 15) transects, and within the dry season. The border of each mesocosm arena was delimited by a mesh fence (approximately 15 cm tall plastic netting), and at the center of each mesocosm, we placed a single 150 g experimental fecal deposit of fresh human feces. Each fecal deposit was mixed with 70 plastic seed mimics in three size classes (1 cm diameter, N = 10, 10 mm diameter, N = 20, 5 mm diameter, N = 40). Seed mimics (rather than real seeds) are an ideal proxy for real seeds in tropical forest, as they are not subject to rodent or ant seed predation or removal and have similar burial rates by beetles [16]. This study design allows dung beetles to freely enter the mesocosm, and engage in the feeding and reproductive activities that translate into feces removal and seed burial, while preventing the removal of brood balls by species with a ‘rolling’ nesting strategy. After a 24-h exposure period to the dung beetle community, we measured the number of seed mimics of each size class buried ≥1 cm under the soil surface. Further details and images of mesocosm setups can be found in Braga et al. (2013).

All necessary permits were obtained for this work from the Brazilian Council Scientific and Technological Development (CNPq) and the Brazilian Institute of Environment and Renewable Natural Resources (IBAMA) under SISBIO permit 16620-1. Collections took place in lands within sustainable development forest reserves, under the jurisdiction of State of Amazonas Secretariat for the Environment and Sustainable Development (SDS-CEUC) and the Chico Mendes Institute of Biodiversity Conservation (ICMBio). No protected species were captured, sampling of all mammal species were restricted to non-invasive line-transect censuses, and no primates, rodents, ungulates (or indeed any mammals at all) were handled during the study.

### Data Analysis

**Figure 2:**
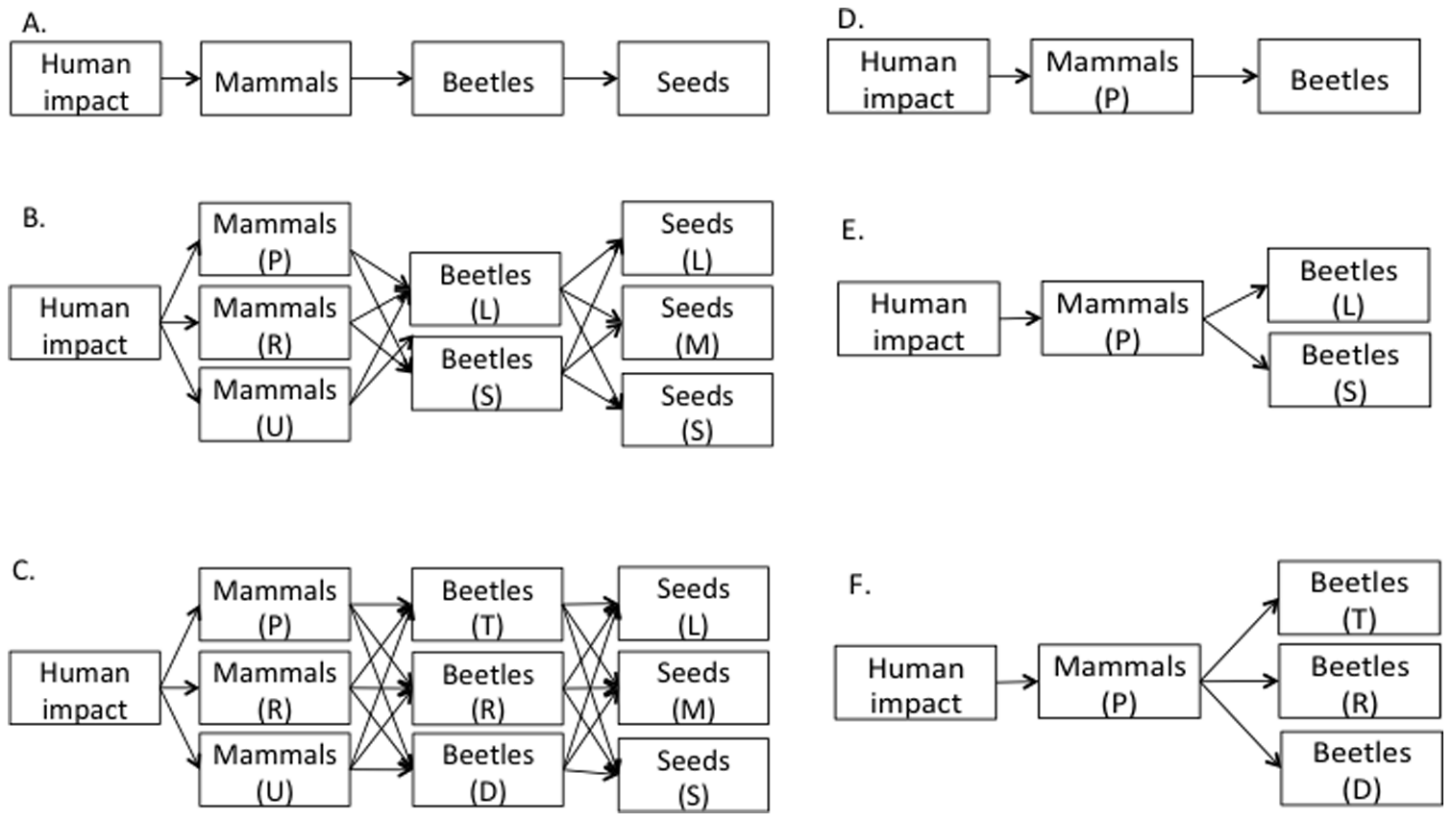
Three alternative trophic cascade structures along the detritus pathway in *terra firme* and *várzea* forest. Cascade structures are represented as either community-level (A, D) or trait-defined (B–F). Mammal abbreviations include: primate (P), rodent (R) and ungulate (U). Dung beetle size classes include: small (S) and large (L). Dung beetle nesting strategies include: tunneler (T), roller (R) and dweller (D).

We examined the evidence for three distinct cascade structures, represented as either community-level (Fig. 2a, d) or trait-defined cascades (Fig. 2b, c, e, f). Each hypothesis was examined with two dung beetle metrics (i.e. species richness and total biomass) and analyzed separately for the two forest types (*terra firme* and *várzea*). We explored all models with both multiple generalized linear mixed models (GLMM) and generalized multilevel path analysis (GMPA) to examine the causal support for overall cascade structure with statistical controls [60].

To examine community-level cascades with generalized multiple linear mixed models, we modeled (i) game mammal abundance as a function of human impact (i.e. settlement size), (ii) dung beetle community species richness or biomass as a function of human impact and game mammal abundance, and (iii) the probability of seed burial as a function of human impact, game mammal abundance and dung beetle species richness or biomass. To discern which dung beetle community attribute (i.e. species richness or biomass) best explained seed burial probability, we compared two separate models including either beetle species richness or biomass with AIC-based model comparison.

To examine trait-defined cascades with a GLMM approach, we re-ran these same models after separating both the dependent and independent variables into groups defined by species traits relevant to trophic interactions. We separated game mammals into broad taxonomic groups (i.e. primate, ungulate and rodent) that both encounter differential selective hunting pressure (e.g. Jerozolimski and Peres 2003) and present distinct feces morphologies and deposition locations (i.e. arboreal vs. terrestrial) [9], [61] for dung beetles. We separated dung beetles into two distinct trait groups based on body mass (i.e. small: <0.1 g and large: ≥0.1 g) and nesting strategies (i.e. roller, tunneler or dweller). For *terra firme* models that included seed burial, we separated seeds into size classes (i.e. small: 2 mm <0.1 g, medium 5 mm: and large: 10 mm). To discern which property of dung beetle community structure (i.e. species richness or biomass) and trait-defined pathway (i.e. body size or nesting strategy) best explained seed burial probability, we compared four separate models for each seed size class with AIC-based model comparison. All analyses used beetle data taken from the 10 traps farthest (≥300 m) from the nearest mesocosm used to measure function to avoid potential bias in beetle capture rates. Ungulates and rodents were excluded from *várzea* forest models due to their rarity in this dataset. All predictors were centered on their means to facilitate interpretation. For all models, we used appropriate error structures (i.e. Poisson or Binomial) and incorporated both season (i.e. wet or dry) and transect identity as random effects in an unreplicated, crossed design [62].

To additionally assess the causal influence of human impact on cascade structure, we used generalized multilevel path models (GMPA) to examine the support for overall cascade structure [60]. GMPA is a generalization of Shipley's d-sep test, wherein a generalized linear mixed model (GLMM) can be used to test a series of related claims of independence in a path diagram. The hypothetical causal structure defined by a DAG can be tested with directional separation tests (d-sep) that quantify if the proposed model corresponds to the patterns of dependence or independence in the data [63]. This d-sep approach involves first finding the ‘basis set’ B_U_ of independence claims implied by a DAG that express the full set of dependence and independence claims implied by the causal graph, when taken as a set (e.g. Table S6). B_U_ is obtained by listing each of the k pairs of variables (X_i_, X_j_) in a causal graph that lack an arrow between them and then conditioning each of those k pairs by the set of variables Z that are either a direct cause of X_i_ or of X_j_ (Table S6). The probability *p_i_* associated with each of the *k* independence claims in B_U_ is obtained using appropriate statistical tests (in our case, GLMMs). The overall hypothesized causal structure implied by the path diagram can then be tested by combining values of *p_i_* using Fisher's test statistic *C* as:
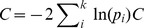
(1)The proposed causal model is rejected if the *P* value associated with the *C* statistic is smaller than the specified α-level (here, α = 0.05) after comparison to a chi-square (χ^2^) distribution with 2*k* degrees of freedom. A significant *P* value supports a rejection of the proposed DAG, as it implies that the data depart significantly from what would be expected under such a causal model [60].

We represented each of our three overall hypotheses about community and trait-defined trophic cascade structure with a DAG (Fig. 2) and tested the *k* independence claims implied by each proposed causal path models (see TS6). These GMPAs used identical model structures as the GLMMs described above. Values of *p_i_* were taken from the *P* value associated with the *t* statistic for the regression coefficient of the composite variable. All analyses were conducted using the ‘nlme’ [64] package in the R environment [65].

## Results

Across the 26 forest transects sampled for both mammals and dung beetles, we conducted a total of 8,430 km of mammal census walks (324±344 km, mean ±1 SD, range 80–1,260 km). These transects were associated with 15 neighboring human settlements, varying in size from five to 500 households (78±25.9 households, median ±1 SD). Human communities adjacent to *terra firme* or *várzea* forest sites were similar in both size (*terra firme* 22.5±29.5 households, *várzea* 19.3±23.3 households, mean ± SD, t_23_ = 0.3, p = 0.76) and distance to censused transects (*terra firme* 0.78±0.39 km, *várzea* 0.84±0.35 km, mean ± SD, t_23_ = −0.38, p = 0.78).

Mammal surveys resulted in observations of 38 species (see Table S1). Primates accounted for 38% of all individuals detected, followed by rodents (18%) and ungulates (16%). Of all species encountered, 58% are considered hunted game species in the region (unpublished data). The mean number of mammal individuals encountered was similar between *terra firme* and *várzea* sites (*terra firme* 1.27±1.4, *várzea* 0.90±0.78 individuals/km, mean ± SD, t_23_ = 0.8, p>0.1), while *terra firme* sites supported significantly higher mammal species richness (*terra firme* 0.015±1.4, *várzea* 0.009±0.78 species/km, mean ± SD, t_22_ = 3.2, p = 0.004).

A total of 10,819 dung beetle individuals in 90 species were captured (*terra firme*: N = 5,887, S = 83; *várzea*: N = 5,513, S = 57; see Table S2). Total beetle abundance per trap was higher in *várzea* forest (*várzea*: 19.5±29.3, *terra firme*: 15.4±15.0 individuals, mean ± SD, t_445_ = −2.26, p = 0.001), while biomass and species richness were higher in *terra firme* (biomass: várzea 0.5±0.41, *terra firme* 0.73±0.53 g, mean ± SD, t_727_ = 509, p<0.001; species richness: *várzea* 5.2±3.3, *terra firme* 6.3±3.9 species, mean ± SD, t_721_ = 4.21, p = 0.001). There were no significant differences in mean individual beetle body mass between forest types (*várzea*: 0.10±0.08 g, *terra firme*: 0.11±0.07 g, mean ± SD, t_664_ = 1.83, p = 0.10). Dung beetle biomass and species richness per trap were significantly higher in the dry season (biomass: dry 2.05±1.87 g, wet 0.89±856 g, mean ± SD, t_560_ = 9.4, p<0.0001; richness: dry 7.0±3.9, wet 3.9±2.4 species, mean ± SD, t_560_ = 11.0, p<0.0001).

Most dung beetle species were diurnal (67%) and used a tunneling nesting strategy (58%). For our analyses, a total of 68 species were classified as ‘small’ (i.e. <0.1 g; 0.022±0.02 g; mean ±1 SD, range 0.0001–0.092 g) and 31 species as ‘large’ (i.e. ≥0.1 g; 0.256±0.187 g; range 0.103–0.79 g). The distribution of nesting strategies across small and large species was similar, with the majority classified as ‘tunnelers’ (small: 64%; large: 68%), while ‘rollers’ represented 30% of small species and 20% of large species. The proportion of seed mimics (hereafter, seeds) removed by detritivores per forest site was greatest for large seeds, lowest for medium seeds and intermediate for small seeds (large 0.22±0.27; medium 0.19±0.18; small 0.20, ±0.13, mean ± SD; n = 15 for each).

### Generalized multiple linear mixed model approach

#### Community-level cascade structure

We found no evidence for community-level cascades in either forest type. Human impact was not associated with community-level mammal abundance (Figure S1, Table S3), dung beetle biomass or species richness (Table S4A, B). Mammal abundance was unrelated with dung beetle biomass and species richness in both *terra firme* and *várzea* forests (Table S4A, B).

The probability of community-level seed burial was equally well explained by models that included beetle biomass or species richness (Table S5A). In neither model was seed burial probability related to human impact, community-level beetle biomass or species richness or mammal abundance (all p>0.05, Table S5B). However, seed burial rate was positively associated with the biomass of small-bodied dung beetles (z_13_ = 4.30, p<0.0001) and ungulate abundance (z_13_ = 6.59, p<0.0001). Seed dispersal was further unrelated to the biomass of large-bodied dung beetles and rodents (all p>0.05) and negatively associated with primate abundance (z_13_ = −3.93, p<0.0001).

### Trait-defined cascade structures

**Figure 3:**
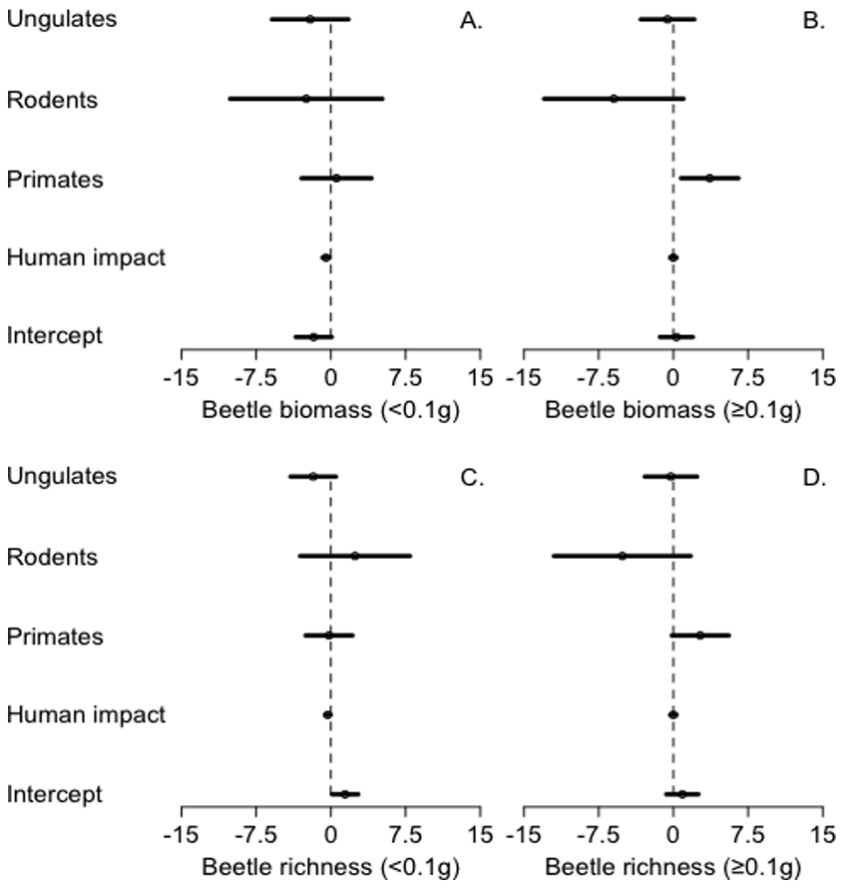
Standardized regression coefficients (β) for GLM models of *terra firme* dung beetle biomass and species richness. Models conducted separately for (A, C) small-bodied and (D, E) large-bodied beetle species; model terms include human impact and game mammal abundance.

Across trait-defined mammal groups, only hunted primates in *terra firme* forest showed a significant negative relationship with human impact (t_13_ = −1.18, p = 0.026; all other p>0.05, see Table S3). Across beetle body-mass defined models in *terra firme* forests, rodent and ungulate abundances were unrelated to beetle body mass (all p>0.05, see Table S3A). In *terra firme* forests, both the biomass and species richness of small beetle species were negatively correlated with human impact (biomass z_244_ = −2.76, p = 0.006; richness z_244_ = −2.50, p = 0.013; Fig. 3A, C), yet remained independent of the abundance of all three mammal groups (all p>0.05, Table S4A). In contrast, the biomass and species richness of large beetle species were independent of human impact (all p>0.05, Fig. 3b,d see Table S4B), yet positively correlated with hunted primate abundance (biomass z_244_ = 3.65, p = 0.013; richness z_244_ = 1.86, p = 0.063; Fig. 3). Both small and large beetles were unrelated to the abundance of hunted rodents and ungulates abundances (all p>0.05, see Table S3A). Neither biomass nor species richness of small beetles in *várzea* forests were associated with human impact or primate abundance (all p>0.05, see Table S4B). Finally, we found strongly positive relationships in *terra firme* forests between both beetle species richness and abundance (r_13_ = 0.95, p<0.0001) and biomass (r_13_ = 0.86, p<0.0001), suggesting the absence of density or biomass compensation.

Across beetle nesting strategy-defined models, the biomass and species richness of all three strategies were independent of human impact in both *terra firme* and várzea forest (all p>0.05, see Table S4). In *terra firme* forest, the biomass of beetles with a ‘dwelling’ strategy was positively correlated with hunted primate abundance (z_244_ = 2.03, p = 0.042). The biomass of species with a ‘roller’ strategy was negatively associated with game rodent abundance (z_244_ = −1.98, p = 0.048), while the species richness of rollers was negatively correlated with ungulate abundance in *terra firme* forest (z_244_ = −2.17, p = 0.03). In *várzea* forest, the abundance of hunted primates was unrelated to all measures of nesting strategy (all p>0.05, see Table S4B).

### Trait-defined detritivore-mediated process rates

**Figure 4:**
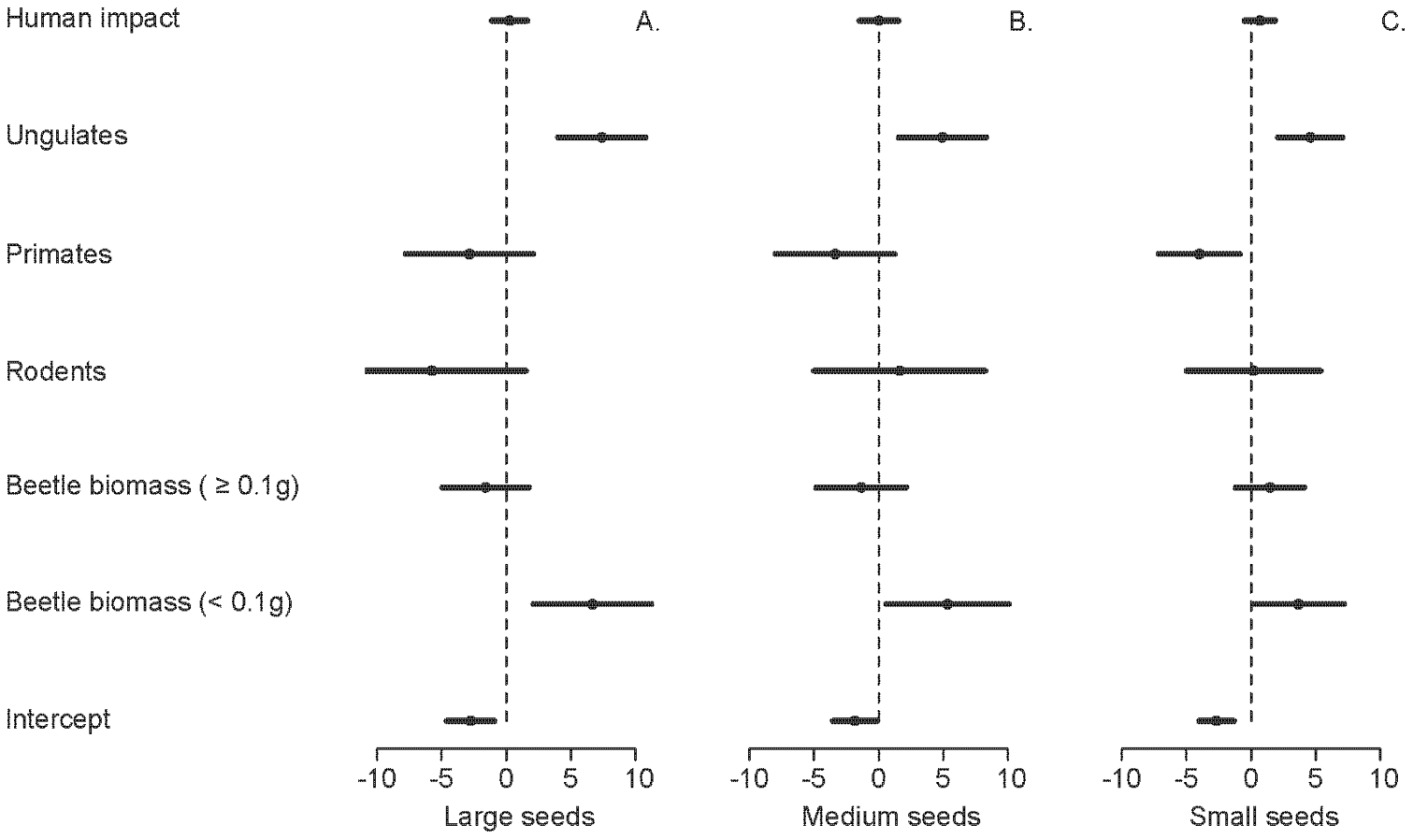
Standardized regression coefficients (β) for GLM models of the probability of secondary seed burial. Model terms include human impact, game mammal abundance and dung beetle biomass.

For all three seed size classes, the top AIC model included the biomass of body-mass-defined trait groups (Table S5). Human impact was unrelated to the probability of seed burial, irrespective of seed size (all p>0.05, Table S5). All classes of seed size showed a strong positive relationship between seed burial probability and the biomass of small-bodied beetles (large: z_15_ = 2.87, p = 0.0041; medium: z_15_ = 2.21, p = 0.027; small: z_15_ = 2.05, p = 0.041), as well as ungulate abundance (large: z_15_ = 4.29, p<0.0001; medium: z_15_ = 2.87, p = 0.004; small: z_15_ = 3.62, p<0.0001). The likelihood of small seed burial was negatively associated with primate abundance (z_59_ = −2.51, p = 0.012, Fig. 4). Rodent abundance was unrelated to seed burial for all size classes (all p> 0.05, see Table S5B).

### Generalized multilevel path analysis approach

**Figure 5:**
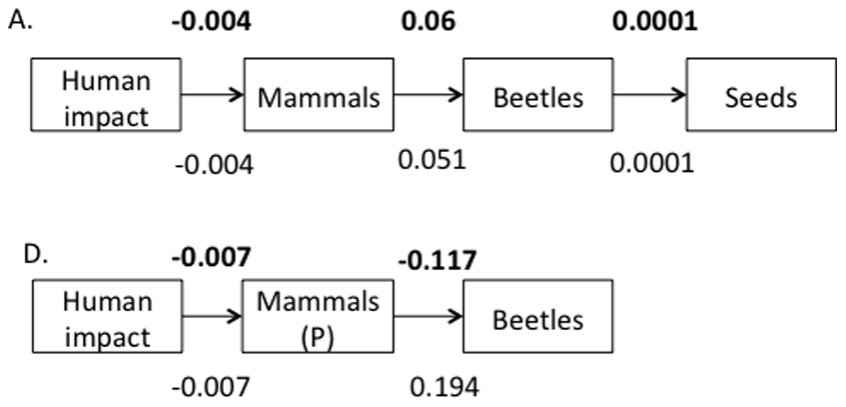
Model structure and standardized path coefficients from generalized multilevel path analysis. Path coefficients (i.e. standardized regression coefficients) above each DAG of the detrital pathway in (A) *terra firme* and (D) *várzea* forests represent values for models based on dung beetle biomass; path coefficients below represent models based on dung beetle species richness. No path coefficients were significant at the α = 0.05 level.

We found support for community-level (Fig. 2a, d), but not trait-defined (Fig. 2b, c, e, f) cascade structure in both *terra firme* and *várzea* forest and for both dung beetle community attributes (*terra firme*: biomass C_6_ = 4.17, p = 0.65, species richness C_6_ = 5.12, p = 0.53; *várzea*: biomass C_2_ = 1.96, p = 0.37, species richness C_2_ = 0.75, p = 0.69). Despite this strong overall model support, individual path coefficients from both forest types were weak (Fig. 5), and there was no evidence of significant relationships between any trophic level in either forest type.

## Discussion

To our knowledge, this represents the first landscape-scale study on the influence of human activity on the structure and function of the fecal detrital pathway. We show that human presence in tropical forests can influence the fecal detritus food web structure via body-size-dependent responses by detritivores. These indirect impacts on fecal detritivores occurred even in the absence of strong community-level responses by game mammals to human activities. Human presence in tropical forest was most strongly associated with declining abundance of large-bodied primate game species, as commonly observed elsewhere in tropical forests [26], [66]. We found no evidence that human activity indirectly influence rates of detritivore-mediated secondary seed dispersal rates. We also found few consistent relationships between beetle-mediated seed dispersal and game mammal abundance. Seed dispersal rates were positively associated with the biomass of small, but not large-bodied beetles – an unexpected finding given the previous empirical support for a dominant role of larger-bodied beetle species in the secondary dispersal of large seeds [16], [58], [67], [68]. Finally, we found stronger associations between dung beetles and both human presence and game mammal abundance in upland *terra firme* forests, compared to seasonally flooded *várzea* forests. Similar to other Neotropical studies, we detected no evidence of density or biomass compensation across the fecal detrivore community [69].

We found no consistent patterns of association between dung beetle nesting strategy and responses to human presence or game mammal abundance. Beetle species with a ‘dwelling’ strategy were positively associated with primate abundance in *terra firme* forest. In the Neotropics, all dwellers are strictly coprophagous members of a single genus (Eurysternus). In contrast, species with a ‘rolling’ nesting strategy were negatively associated with the abundance of both caviomorph rodents (i.e. *Dasyprocta fuliginosa, Agouti paca*) and ungulates (i.e. *Mazama spp., Tapirus terrestris, Tayassu pecari, Pecari tajacu*). While the predation of adult dung beetles by caviomorph rodents and ungulates is a possible explanation for these results [70], future manipulative studies will be required to understand the mechanisms behind these observed beetle-mammal associations.

We found stronger support for trophic effects defined by dung beetle body-size than those defined by nesting strategy. Contrary to our initial expectations [9], small-bodied beetle species were disproportionately sensitive to human presence in (upland) tropical forests. A similarly negative effect on small-bodied beetles was reported by Andresen and Laurance (2007) in Barro Colorado Island, Panama, even 15 years after hunting activity had ceased [11]. Culot et al. (2013) also reported that negative trends between mammal abundance and dung beetle species richness were stronger for smaller-bodied beetles [71]. In contrast, human impact was unrelated to the diversity and biomass larger-bodied beetles in either forest type. The combination of hunting pressure, and mammal avoidance near permanent human settlement may translate into ‘sinks’ of lower feces availability that disproportionately impact smaller beetle species with reduced dispersal abilities that are incapable of subsidizing their diet in neighboring patches with greater fecal resources. In contrast, the neutral response by large beetles to human activity may be a result of three potentially interacting phenomena: (1) dispersal-mediated buffering, (2) resource scarcity effects, and/or (3) the human subsidy effect.

First, the high vagility of large dung beetles may enable the detection and pursuit of fecal resources across wider spatial extents [72], which can buffer the fitness effects of local resource scarcity, relative to smaller beetles. Such effects may be more likely at early stages of defaunation, when local resource depletion operates in patches of higher and lower resources, rather than across an entire region.

Alternatively, the neutral abundance response of larger-bodied beetles in areas of human settled areas may arise from a positive relationship between local resource scarcity and capture rates [73], [74]. Such resource scarcity effects are a common practical issue for field studies that use attractive baits [75], are more likely to influence capture rates of larger individuals with elevated dispersal abilities, responding to food resources detected over wider spatial scales. Future work should attempt to determine the role of such scarcity effects on observational studies that report perplexingly strong and negative relationships between beetle abundance and mammal biomass [71]. Third and finally, local subsidies of human fecal resources may positively influence either the population density or observed capture rates of larger-bodied beetles, given the ready attraction of dung beetles to primate (including human) feces, and the frequent occurrence of open-air defecation in this study system (Nichols, pers. obs.). We consider this final alternative hypothesis to be relatively unlikely, both because a human fecal subsidy should positively influence small and large-bodied beetles, and because the positive impact of this subsidy should be tempered by the strongly negative abundance responses to the agricultural land-use that surround human settlements [76].

For large-bodied dung beetles in *terra firme* forest, we observed a decoupled response to human activity (neutral) and the abundance of large-bodied ateline primates (positive) that were themselves strongly impacted by human activity. This decoupled response may reflect the high demographic consequences of dispersal through the faunal depletion zone for large-bodied primates [38], [77], [78]; effects that may be relatively neutral for large-bodied beetles.

Any of these three of these size-biased processes may translate into a degree of functional ‘spatial insurance’ [72] across the heterogeneous fecal resource landscape of hunted tropical forests. The persistence of such spatial insurance is likely to be sensitive to activities that reduce connectivity between fecal resource patches, including increased mammal offtake around permanent human settlements and localized land-use changes following agricultural expansion. Taken together, these results suggest that the lasting human footprint on dung beetle persistence may accrue through two distinct pathways: early declines of small-bodied beetle species, compounded by subsequent declines of large-bodied specialists.

We found no relationship between human activity in tropical forest and detritivore-mediated rates of secondary seed dispersal. Strong attenuation between top-down forces and processes related to plant growth and demography are not uncommon in trophic cascade studies, particularly those that focus on density-mediated indirect interactions between predators and plants [79], [80]. We also found a strong, unexpected relationship between the biomass of small-bodied beetle species and the probability of burial for large seeds. These results differ from those reported from an identical experimental design in another western Amazonian site, where both the biomass and richness of larger-bodied beetles and richness (though not biomass) of small beetles were positively associated with large seed burial rate [81]. These contradictory results echo a lack of clear, consistent associations between dung beetle community attributes and beetle-mediated ecological function in observational studies [67], [74], [82].

We posit that part of this variability may arise from a widespread sampling artifact. In studies that collect data on ecological process rates and biodiversity in separate steps (i.e. through mesocosms and pitfall traps, as used here), differences in the size of the baits used to measure function (150 g) or attract beetle diversity in pitfalls (20 g) may introduce a large-beetle bias in ecological process measurements, as larger-bodied beetles can respond from greater distances to the larger scent plumes emitted by functional mesocosms. Horgan (2005) reported marked differences in observed beetle-function relationships when mesocosm data were compared with biodiversity data collected either directly within the mesocosms, or in neighboring pitfall traps (as used here). This suggests that size-biased sampling artifacts can indeed influence the observed correlations between dung beetle community attributes and function. This artifact can be overcome with simple modifications to the collection of either paired functional or biodiversity data.

We also found that forest type strongly mediated trophic cascade strength, with clearer evidence for human impact-induced cascade effects in *terra firme*, rather than *várzea* forests. These differences were strong, and are likely associated with the biological and socio-economic features unique to each forest types, despite their naturally close spatial proximity. Biomass of all vertebrates [83] and large-bodied primates in particular [84] can be orders of magnitude lower in upland, oligotrophic forests than in neighboring seasonally flooded forests [30], potentially leaving stronger signatures of co-decline in areas where hunting pressure was recently or historically high. In addition, as opportunistic hunting typically accompanies extractive and agricultural activities [45], [85], the year-round accessibility of *terra firme* forests, and location of manioc fields within *terra firme* sites may support the exploitation of large-bodied primates in upland forest, beyond the threshold at which active pursuit is predicted on the basis of hunting effort alone [86]. These general differences between *terra firme* and *várzea* forests were specifically present across our study region, where the two forest types differed in mean basal area [34], vertebrate community structure (this study; Endo unpublished) and patterns of human forest access and use [87], [88].

Finally, we found that our use of two analytical approaches (i.e. GLMM and GMPA) resulted in strikingly different assessments of both cascade structure and strength. Path analysis suggested that a community-level model of cascade structure was most appropriate for both forest types, a result potentially linked to the higher number of parameters in trait-defined models (see Table S6). Despite the support for these community models, the individual path coefficients linking trophic levels were weak, reflecting similar results to those reported from generalized linear mixed models. Taken together, these results highlight the complementarity of these analytical approaches, and suggest that further examination of the structure of trophic cascades along the fecal detritus pathway is warranted.

Our study also raises an interesting and currently underexplored issue, related to the net effects on ecological function of cascade dynamics in donor-controlled systems. When the indirect interactions that lead to detritivore decline are mediated by a reduction in the availability of detrital resources, and the ecological processes of interest result from the consumption of those detrital resources, does biodiversity loss beget a true net loss of ecological functioning? This question stems from the donor-controlled nature of the detritus pathway, and is generalizable to any detritus based system. While feedbacks between plants and plant consumers in green-world pathways largely determine overall cascade structure and strength [17], [89], the absence of interactions between detritus and detritus consumers has been alternatively proposed to *weaken* detrital cascades [90] or alternatively *strengthen* them relative to plant cascades, by tightly coupling consumption to resource depletion. A quantitative exploration of these questions will demand new information on the topology of ecological interaction networks formed by fecal detritus producers and consumers, as well as an improved understanding of the spatial dynamics of feces producer and consumer co-decline.

Our landscape-level study allowed us to detect evidence for a cascading impact of human activity on detritivores, but not detritivore-mediated processes [91]. Future manipulated experiments will be necessary to determine the mechanisms by which human activity influences cascade structure and strength in the fecal detritus web. In particularly, disentangling the potential influence of dung beetle mobility on cascade dynamics observed here will require additional attention to the spatial dimensions of trophic cascades [92], including spatial patterns of mammal defaunation [93], the interaction of beetle dispersal ability and fecal resource availability [94], and how spatial exchanges across areas exposed to variable levels of hunting pressure may affect dung beetle-mediated ecological process rates. Recent evidence also suggests that future inclusion of trait-mediated indirect interactions in cascade studies will be critical to understanding cascade dynamics in the fecal detritus systems [10]. Such mechanisms may include predator-mediated changes in detritivore behavior, physiology or even stoichiometry [95], [96], and importantly may strongly influence the observed functional and spatial relationships [97] between detritivores and plant-facilitating processes, even in the absence of obvious density-mediated effects [10]. Our findings provide the first landscape-scale evidence that human presence in tropical forests can influence the structure of fecal detritus pathways and support the ongoing prioritization of research that explores the impacts of human use of tropical forest on food web structure and function.

## Supporting Information

Figure S1 **Relationship between human settlement size and the abundance of large and medium diurnal tropical mammals.**
(TIFF)

Table S1 **Summary of mammals encountered through 8,430 km of line transect sampling in both** ***várzea*** **and** ***terra firme*** **forest sites (** ***terra firme*** **N = 15,** ***várzea*** **N = 11).**
(DOCX)

Table S2 **Total number of captures per habitat type and values of three species traits: mean body mass, activity period and food relocation strategy.** Abundances represent total captures from *terra firme* (n = 369 trap nights) and *várzea* forests (n = 277 trap nights).
(DOCX)

Table S3 **Generalized linear regression results of medium and large-bodied diurnal game mammals as a function of human impact in** ***terra firme*** **and** ***várzea*** **forest.**
(DOCX)

Table S4 **Generalized linear regression results of detritivorous dung beetles as a function of human impact, game mammal abundance and sampling season in** ***terra firme*** **(A) and** ***várzea*** **forest (B).**
(DOCX)

Table S5 **AIC model selection results (A) and generalized linear regression results (B) for AIC top models of dung beetle-mediated secondary seed burial.**
(DOCX)

Table S6 **The Bu basis set of d-separation for three alternative causal path models of the direct and indirect impacts of human activity in** ***terra firme*** **and** ***várzea*** **forests.**
(DOCX)
