# Supplementary material for: Human-Induced Trophic Cascades along the Fecal Detritus Pathway
Source: PLoS One. 2013 Oct 16;8(10):e75819. doi: 10.1371/journal.pone.0075819 (PMC3797778; doi:10.1371/journal.pone.0075819)
Supplement: Table S1 — Summary of mammals encountered through 8,430 km of line transect sampling in both várzea and terra firme forest sites ( terra firme N = 15, várzea N = 11). (DOCX) [file pone.0075819.s002.docx]

| Supplementary Table 1. Summary of mammals encountered through 8,430km of line transect sampling in both várzea and *terra firme* forest sites (*terra firme* N=15, várzea N=11)*.* All data collected between January 2008 and December 2010 in the Médio Juruá Extractive Reserve and the Uacari Sustainable Development Reserve, Amazonas State, Brazil. | | | | |
| --- | --- | --- | --- | --- |
| Trait | Target game species? | Mammal | Individuals km^-1^ |  |
| Group |  | Species |  |  |
| Dasypodid | YES | *Dasypus spp (novemcinctus* and *kappleri)* | 0.2 |  |
| Dasypodid | YES | *Priodontes maximus* | 0.2 |  |
| Didelphid | NO | *Didelphis marsupialis* | 0.6 |  |
| Felid | NO | *Leopardus pardalis* and *wiedii* | 0.2 |  |
| Felid | NO | *Panthera onca* | 0.2 |  |
| Felid | NO | *Puma concolor* | 0.2 |  |
| Mustelid | NO | *Eira barbara* | 0.4 |  |
| Mustelid | NO | *Lontra longicaudis* | 0.2 |  |
| Myrmecophagid | NO | *Myrmecophaga tridactyla* | 0.2 |  |
| Myrmecophagid | NO | *Tamandua tetradactyla* | 0.3 |  |
| Primate | NO | *Aotus (nigriceps and nancymaae)* | 0.8 |  |
| Primate | NO | *Cacajao calvus* | 7.8 |  |
| Primate | NO | *Callicebus (cupreus, regulus* and *purinus)* | 0.8 |  |
| Primate | NO | *Cebuella pygmaea* | 0.9 |  |
| Primate | NO | *Cebus albifrons* | 4.8 |  |
| Primate | NO | *Pithecia spp (monachus, irrorata* and possibly *albicans)* | 1 |  |
| Primate | NO | *Saguinus mystax* | 2 |  |
| Primate | NO | *Saguinus spp and sciureus* | 1.9 |  |
| Primate | NO | *Saimiri (sciureus* and *boliviensis)* | 6.8 |  |
| Primate | YES | *Alouatta seniculus* | 1.3 |  |
| Primate | YES | *Ateles chamek* | 2.3 |  |
| Primate | YES | *Sapajus apella* | 1.7 |  |
| Primate | YES | *Lagothrix spp (cana* and *poeppigii)* | 4.6 |  |
| Procyonid | NO | *Nasua nasua* | 1.8 |  |
| Rodent | NO | *Coendou prehensilis* | 0.2 |  |
| Rodent | NO | *Microsciurus flaviventer* | 0.2 |  |
| Rodent | NO | *Sciurus spp* | 0.3 |  |
| Rodent | YES | *Agouti paca* | 0.2 |  |
| Rodent | YES | *Dasyprocta fuliginosa* | 0.2 |  |
| Rodent | YES | *Hydrochoerus hydrochaeris* | 0.6 |  |
| Rodent | YES | *Myoprocta pratti* | 0.2 |  |
| Ungulate | YES | *Mazama americana* | 0.2 |  |
| Ungulate | YES | *Mazama nemorivaga* | 0.2 |  |
| Ungulate | YES | *Mazama spp (americana* or *nemorivaga)* | 0.2 |  |
| Ungulate | YES | *Pecari tajacu* | 0.9 |  |
| Ungulate | YES | *Tapirus terrestris* | 0.3 |  |
| Ungulate | YES | *Tayassu pecari* | 6.8 |  |
