# Supplementary material for: Human-Induced Trophic Cascades along the Fecal Detritus Pathway
Source: PLoS One. 2013 Oct 16;8(10):e75819. doi: 10.1371/journal.pone.0075819 (PMC3797778; doi:10.1371/journal.pone.0075819)
Supplement: Table S2 — Total number of captures per habitat type and values of three species traits: mean body mass, activity period and food relocation strategy. Abundances represent total captures from terra firme (n = 369 trap nights) and várzea forests (n = 277 trap nights). (DOCX) [file pone.0075819.s003.docx]

Supplementary Table 2. Total captures per habitat type, and values of three species traits: mean body mass (grams), activity period (N: nocturnal; D: diurnal) and food relocation strategy (R: roller, T: tunneler). Abundance totals represent total captures from a total of 369 trap nights in *terra firme* forest and 277 trap nights in *várzea* forests.

|  | Species | N *terra firme* | N *várzea* | Mean body mass (g) | Activity period | Food relocation |
| --- | --- | --- | --- | --- | --- | --- |
| 1 | *Anisocanthon aff sericinus* | 2 | 124 | 12.97 | na | R |
| 2 | *Ateuchus aff murrayi* | 177 | 10 | 8.66 | N | T |
| 3 | *Ateuchus aff. candezei* | 3 | 0 | 24.47 | N | T |
| 4 | *Ateuchus aff. ovalis* | 1 | 0 | 8.25 | N | T |
| 5 | *Ateuchus connexus* | 51 | 0 | 25.64 | N | T |
| 6 | *Ateuchus scatimoides* | 5 | 1 | 10.45 | N | T |
| 7 | *Ateuchus sp1* | 3 | 0 | 9.61 | N | T |
| 8 | *Ateuchus sp2* | 34 | 0 | 19.07 | D | T |
| 9 | *Canthidium aff collare* | 2 | 22 | 13.23 | D | T |
| 10 | *Canthidium aff cupreum* | 22 | 0 | 16.33 | D | T |
| 11 | *Canthidium aff lentum* | 4 | 71 | 27.64 | D | T |
| 12 | *Canthidium onitoides* | 160 | 68 | 50.37 | D | T |
| 13 | *Canthidium sp1* | 18 | 3 | 17.01 | D | T |
| 14 | *Canthidium sp10* | 2 | 0 | 2.00 | D | T |
| 15 | *Canthidium sp2* | 6 | 33 | 9.82 | D | T |
| 16 | *Canthidium sp3* | 3 | 0 | 25.30 | D | T |
| 17 | *Canthidium sp4* | 214 | 1 | 9.125 | D | T |
| 18 | *Canthidium sp5* | 0 | 3 | 10.00 | D | T |
| 19 | *Canthidium sp6* | 0 | 5 | 5.36 | D | T |
| 20 | *Canthidium sp7* | 0 | 5 | 0.80 | D | T |
| 21 | *Canthidium sp8* | 2 | 0 | 14.32 | D | T |
| 22 | *Canthidium splendidus* | 210 | 32 | 12.79 | D | na |
| 23 | *Canthon aff angustatus1* | 1 | 0 | 8.73 | D | R |
| 24 | *Canthon aff angustatus2* | 1 | 3 | 7.93 | D | R |
| 25 | *Canthon aff quinquemaculatus* | 34 | 349 | 38.99 | D | R |
| 26 | *Canthon aff sericatus* | 0 | 2 | 8.10 | D | R |
| 27 | *Canthon aff smargardulus* | 135 | 0 | 138.96 | D | R |
| 28 | *Canthon bimaculatus* | 4 | 0 | 28.50 | D | R |
| 29 | *Canthon coloratus* | 1 | 0 | 21.625 | D | R |
| 30 | *Canthon fulgidus* | 58 | 0 | 103.34 | D | R |
| 31 | *Canthon luteicollis* | 245 | 1 | 57.64 | D | R |
| 32 | *Canthon proseni* | 246 | 314 | 92.255 | D | R |
| 33 | *Canthon quadriguttatus* | 3 | 0 | 20.50 | D | R |
| 34 | *Canthon rufocoeruleus* | 0 | 616 | 4.91 | D | R |
| 35 | *Canthon semiopacus* | 72 | 0 | 43.515 | D | R |
| 36 | *Canthon triangularis* | 246 | 1346 | 92.03 | D | R |
| 37 | *Coprophanaeus callegarii* | 3 | 0 | 153.52 | C | T |
| 38 | *Coprophanaeus ensifer* | 1 | 0 | 2754.2 | na | na |
| 39 | *Coprophanaeus telamon* | 30 | 1 | 453.84 | D | T |
| 40 | *Deltochilum aff peruanum* | 61 | 208 | 68.59 | C | R |
| 41 | *Deltochilum aff septemstriatum* | 29 | 0 | 44.26 | C | R |
| 42 | *Deltochilum aff sericeum* | 50 | 0 | 106.86 | C | R |
| 43 | *Deltochilum amazonicum* | 81 | 17 | 496.38 | N | R |
| 44 | *Deltochilum orbiculare* | 41 | 0 | 300.10 | N | R |
| 45 | *Dichotomius aff lucasi* | 75 | 0 | 105.10 | N | T |
| 46 | *Dichotomius aff. fortestriatus* | 265 | 18 | 113.77 | D | T |
| 47 | *Dichotomius apicalis* | 1 | 0 | 146.45 | N | T |
| 48 | *Dichotomius mamillatus* | 47 | 12 | 461.79 | N | T |
| 49 | *Dichotomius melzeri* | 2 | 0 | 529.90 | N | T |
| 50 | *Dichotomius nimuendaju* | 2 | 0 | 550.70 | N | T |
| 51 | *Dichotomius ohausi* | 7 | 1 | 162.90 | N | T |
| 52 | *Dichotomius prietoi* | 167 | 4 | 531.35 | N | T |
| 53 | *Dichotomius robustus* | 2 | 0 | 119.00 | N | T |
| 54 | *Dichotomius sp1* | 1 | 0 | 186.30 | N | T |
| 55 | *Dichotomius worontzowi* | 4 | 1 | 165.48 | N | T |
| 56 | *Eurysternus caribaeus* | 323 | 22 | 117.35 | D | D |
| 57 | *Eurysternus hamaticolis* | 47 | 81 | 185.16 | D | D |
| 58 | *Eurysternus wittmerorum* | 211 | 34 | 23.48 | na | D |
| 59 | *Eurysterus cayennensis* | 894 | 18 | 29.36 | D | D |
| 60 | *Eurysterus foedus* | 34 | 6 | 154.32 | D | D |
| 61 | *Eurysterus hypocrita* | 276 | 16 | 189.95 | D | D |
| 62 | *Eurysterus strigilatus* | 22 | 6 | 13.16 | D | D |
| 63 | *Eurysterus vastiorum* | 6 | 7 | 13.53 | D | D |
| 64 | *Genera nova* | 0 | 114 | 5.85 | na | na |
| 65 | *Gromphas amazonica* | 1 | 56 | 156.88 | na | T |
| 66 | *Ontherus pubens* | 8 | 14 | 76.97 | N | T |
| 67 | *Onthophagus aff bidentatus* | 276 | 263 | 8.85 | D | T |
| 68 | *Onthophagus aff clypeatus* | 3 | 0 | 13.36 | D | T |
| 69 | *Onthophagus aff haemathopus* | 450 | 40 | 16.55 | D | T |
| 70 | *Onthophagus marginicollis* | 1 | 1 | 12.76 | D | T |
| 71 | *Onthophagus onorei* | 147 | 0 | 4.03 | D | T |
| 72 | *Oxysternon conspicillatum* | 11 | 0 | 789.62 | D | T |
| 73 | *Oxysternon lautum* | 27 | 345 | 596.07 | D | T |
| 74 | *Oxysternon silenus zikani* | 17 | 3 | 135.38 | D | T |
| 75 | *Phanaeus cambeforti* | 22 | 3 | 142.55 | D | T |
| 76 | *Phanaeus chalcomelas* | 89 | 0 | 264.26 | D | T |
| 77 | *Pseudocanthon aff xanthurus* | 0 | 1 | 3.15 | D | na |
| 78 | *Pseudocanthon sp 1* | 1 | 3 | 5.05 | D | na |
| 79 | *Scybalocanthon sp1* | 39 | 0 | 42.17 | D | na |
| 80 | *Scybalocanthon sp2* | 64 | 6 | 25.98 | D | na |
| 81 | *Scybalocanthon sp3* | 9 | 86 | 26.65 | D | R |
| 82 | *Scybalocanthon sp4* | 13 | 697 | 25.44 | D | R |
| 83 | *Scybalocanthon sp5* | 2 | 0 | 5.10 | D | R |
| 84 | *Sylvicanthon aff bridarollii* | 7 | 146 | 44.83 | D | R |
| 85 | *Sylvicanthon sp2* | 6 | 1 | 22.45 | D | R |
| 86 | *Uroxys sp1* | 9 | 6 | 7.18 | D | T |
| 87 | *Uroxys sp2* | 2 | 23 | 0.10 | D | T |
| 88 | *Uroxys sp3* | 14 | 140 | 2.62 | D | T |
| 89 | *Uroxys sp4* | 1 | 2 | 0.10 | D | T |
| 90 | *Uroxys sp5* | 21 | 102 | 2.75 | D | T |
