# Supplementary material for: Human-Induced Trophic Cascades along the Fecal Detritus Pathway
Source: PLoS One. 2013 Oct 16;8(10):e75819. doi: 10.1371/journal.pone.0075819 (PMC3797778; doi:10.1371/journal.pone.0075819)
Supplement: Table S3 — Generalized linear regression results of medium and large-bodied diurnal game mammals as a function of human impact in terra firme and várzea forest. (DOCX) [file pone.0075819.s004.docx]

Supplementary Table 3. Results of generalized linear regressions relating mammal abundance communities as a function of human impact in *terra firme* and várzea forest. All data collected between January 2008 and December 2010 in the Medio Jurua Extractive Reserve and the Uacari Sustainable Development Reserve, Amazonas State, Brazil.

| Model | Coefficient | | Est. | SE | *t* | *p-value* | Lower 95% CI | Upper 95% CI |
| --- | --- | --- | --- | --- | --- | --- | --- | --- |
| *Terra firme forest* | | |  |  |  |  |  |  |
| Community-level | | (Intercept) | -0.39 | 0.28 | -1.39 | 0.165 | 0.37 | -1.14 |
| Community-level | | Human impact | -0.58 | 0.44 | -1.32 | 0.188 | 0.55 | -1.71 |
| Game primate | | (Intercept) | -1.83 | 0.32 | -5.70 | 0.000 | 1.76 | -5.42 |
| Game primate | | Human impact* | -1.18 | 0.53 | -2.23 | 0.026 | 1.13 | -3.49 |
| Game rodent | | (Intercept) | -3.55 | 0.36 | -9.85 | 0.000 | 3.40 | -10.50 |
| Game rodent | | Human impact | -0.11 | 0.59 | -0.18 | 0.854 | 0.10 | -0.32 |
| Ungulate | | (Intercept) | -3.14 | 0.38 | -8.30 | 0.000 | 3.02 | -9.31 |
| Ungulate | | Human impact | -0.91 | 0.61 | -1.51 | 0.131 | 0.88 | -2.71 |
| Non-game primate | | (Intercept) | -1.10 | 0.32 | -3.46 | 0.001 | 1.06 | -3.26 |
| Non-game primate | | Human impact | -0.45 | 0.50 | -0.89 | 0.375 | 0.43 | -1.32 |
| Non-game rodent | | (Intercept) | -4.57 | 0.38 | -11.94 | 0.000 | 4.39 | -13.54 |
| Non-game rodent | | Human impact | -0.14 | 0.67 | -0.21 | 0.838 | 0.13 | -0.41 |
| *Várzea forest* | |  |  |  |  |  |  |  |
| Community-level | | (Intercept) | -0.41 | 0.26 | -1.56 | 0.119 | 0.39 | -1.21 |
| Community-level | | Human impact | -0.66 | 0.76 | -0.86 | 0.388 | 0.63 | -1.94 |
| Game primate | | (Intercept) | -1.68 | 0.18 | -9.43 | 0.000 | 1.61 | -4.97 |
| Game primate | | Human impact | -1.00 | 0.55 | -1.83 | 0.068 | 0.96 | -2.95 |
| Non-game primate | | (Intercept) | -1.06 | 0.33 | -3.24 | 0.001 | 1.02 | -3.13 |
| Non-game primate | | Human impact | -0.44 | 0.52 | -0.84 | 0.400 | 0.42 | -1.30 |
| Ungulate | | (Intercept) | -2.97 | 0.42 | -7.03 | 0.000 | 2.86 | -8.80 |
| Ungulate | | Human impact | -0.76 | 0.67 | -1.14 | 0.256 | 0.73 | -2.25 |
| Game rodent | | (Intercept) | -3.25 | 0.39 | -8.24 | 0.000 | 3.12 | -9.61 |
| Game rodent | | Human impact | -0.30 | 0.67 | -0.45 | 0.650 | 0.29 | -0.90 |
