# Supplementary material for: Human-Induced Trophic Cascades along the Fecal Detritus Pathway
Source: PLoS One. 2013 Oct 16;8(10):e75819. doi: 10.1371/journal.pone.0075819 (PMC3797778; doi:10.1371/journal.pone.0075819)
Supplement: Table S4 — Generalized linear regression results of detritivorous dung beetles as a function of human impact, game mammal abundance and sampling season in terra firme (A) and várzea forest (B). (DOCX) [file pone.0075819.s005.docx]

Supplementary Table 4A. Results of generalized linear regressions relating dung beetle communities as a function of human impact, and game mammal abundance in *terra firme* forest. All data collected between January 2008 and December 2010 in the Medio Jurua Extractive Reserve and the Uacari Sustainable Development Reserve, Amazonas State, Brazil.

| Model | Coefficient | Est. | SE | *z* | *p-value* | Lower 95% CI | Upper 95% CI |
| --- | --- | --- | --- | --- | --- | --- | --- |
| \| Community-level biomass \| \| --- \| | (Intercept) | 0.38 | 0.26 | 1.45 | 0.147 | -0.36 | 1.12 |
|  | Mammal abundance | 0.34 | 0.41 | 0.82 | 0.411 | -0.32 | 0.99 |
|  | Human impact | -0.21 | 0.15 | -1.40 | 0.163 | 0.20 | -0.61 |
| Community-level richness | (Intercept) | 1.80 | 0.25 | 7.11 | 0.000 | -1.73 | 5.32 |
|  | Mammal abundance | 0.19 | 0.33 | 0.60 | 0.552 | -0.19 | 0.57 |
|  | Human impact | -0.20 | 0.12 | -1.75 | 0.080 | 0.19 | -0.60 |
| Dweller species biomass | (Intercept) | -0.01 | 1.34 | -0.01 | 0.994 | 0.01 | -0.03 |
|  | Rodent abundance | -5.39 | 5.82 | -0.93 | 0.354 | 5.17 | -15.95 |
|  | Primate abundance* | 5.20 | 2.56 | 2.03 | 0.042 | -4.99 | 15.38 |
|  | Ungulate abundance | -0.89 | 2.39 | -0.37 | 0.711 | 0.85 | -2.62 |
|  | Human impact | -0.44 | 0.28 | -1.59 | 0.112 | 0.42 | -1.29 |
| Roller species biomass | (Intercept) | -2.37 | 1.20 | -1.98 | 0.048 | 2.27 | -7.01 |
|  | Rodent abundance* | -10.31 | 5.22 | -1.98 | 0.048 | 9.89 | -30.50 |
|  | Primate abundance | 2.55 | 2.02 | 1.26 | 0.206 | -2.45 | 7.55 |
|  | Ungulate abundance | -0.90 | 1.94 | -0.46 | 0.645 | 0.86 | -2.65 |
|  | Human impact | -0.08 | 0.22 | -0.37 | 0.714 | 0.08 | -0.24 |
| Tunneler species biomass | (Intercept) | -0.31 | 0.74 | -0.42 | 0.673 | 0.30 | -0.93 |
|  | Rodent abundance | -2.81 | 3.02 | -0.93 | 0.352 | 2.70 | -8.32 |
|  | Primate abundance | 1.96 | 1.24 | 1.58 | 0.114 | -1.88 | 5.81 |
|  | Ungulate abundance | -0.77 | 1.22 | -0.63 | 0.527 | 0.74 | -2.28 |
|  | Human impact | 0.02 | 0.14 | 0.13 | 0.898 | -0.02 | 0.05 |
| Dweller species richness | (Intercept) | 1.25 | 0.91 | 1.38 | 0.166 | -1.20 | 3.71 |
|  | Rodent abundance | -0.07 | 3.93 | -0.02 | 0.985 | 0.07 | -0.22 |
|  | Primate abundance | 1.92 | 1.69 | 1.13 | 0.257 | -1.84 | 5.67 |
|  | Ungulate abundance | -0.71 | 1.55 | -0.46 | 0.646 | 0.68 | -2.11 |
|  | Human impact | -0.20 | 0.18 | -1.11 | 0.269 | 0.19 | -0.59 |
| Roller species richness | (Intercept) | -0.38 | 0.51 | -0.74 | 0.460 | 0.36 | -1.12 |
|  | Rodent abundance | -4.14 | 2.25 | -1.84 | 0.065 | 3.97 | -12.25 |
|  | Primate abundance | 1.34 | 0.95 | 1.40 | 0.160 | -1.28 | 3.96 |
|  | Ungulate abundance* | -2.25 | 1.04 | -2.17 | 0.030 | 2.16 | -6.67 |
|  | Human impact | -0.10 | 0.10 | -1.01 | 0.312 | 0.10 | -0.31 |
| Tunneler species richness | (Intercept) | 1.06 | 0.72 | 1.47 | 0.142 | -1.01 | 3.13 |
|  | Rodent abundance | -0.62 | 2.83 | -0.22 | 0.827 | 0.59 | -1.83 |
|  | Primate abundance | 0.79 | 1.19 | 0.66 | 0.507 | -0.76 | 2.34 |
|  | Ungulate abundance | -0.73 | 1.13 | -0.64 | 0.521 | 0.70 | -2.15 |
|  | Human impact | -0.20 | 0.13 | -1.58 | 0.113 | 0.19 | -0.59 |
| Biomass species < 0.1g | (Intercept) | -1.73 | 0.92 | -1.89 | 0.059 | 1.66 | -5.12 |
|  | Rodent abundance | -2.48 | 3.90 | -0.64 | 0.525 | 2.38 | -7.34 |
|  | Primate abundance | 0.56 | 1.79 | 0.31 | 0.757 | -0.53 | 1.64 |
|  | Ungulate abundance | -2.08 | 1.96 | -1.06 | 0.288 | 1.99 | -6.15 |
|  | Human impact* | -0.49 | 0.18 | -2.76 | 0.006 | 0.47 | -1.44 |
| Biomass species ≥ 0.1g | (Intercept) | 0.30 | 0.84 | 0.35 | 0.724 | -0.29 | 0.88 |
|  | Rodent abundance | -5.98 | 3.57 | -1.68 | 0.094 | 5.74 | -17.69 |
|  | Primate abundance* | 3.65 | 1.47 | 2.48 | 0.013 | -3.50 | 10.80 |
|  | Ungulate abundance | -0.59 | 1.37 | -0.43 | 0.668 | 0.56 | -1.73 |
|  | Human impact | -0.01 | 0.16 | -0.06 | 0.951 | 0.01 | -0.03 |
| Richness species < 0.1g | (Intercept) | 1.42 | 0.67 | 2.12 | 0.034 | -1.36 | 4.21 |
|  | Rodent abundance | 2.42 | 2.81 | 0.86 | 0.390 | -2.32 | 7.15 |
|  | Primate abundance | -0.19 | 1.20 | -0.16 | 0.874 | 0.18 | -0.57 |
|  | Ungulate abundance | -1.78 | 1.16 | -1.53 | 0.126 | 1.71 | -5.26 |
|  | Human impact | -0.32 | 0.13 | -2.50 | 0.013 | 0.30 | -0.94 |
| Richness species ≥ 0.1g | (Intercept) | 0.90 | 0.82 | 1.09 | 0.277 | -0.86 | 2.65 |
|  | Rodent abundance | -5.13 | 3.50 | -1.47 | 0.142 | 4.93 | -15.19 |
|  | Primate abundance | 2.70 | 1.46 | 1.86 | 0.063 | -2.59 | 8.00 |
|  | Ungulate abundance | -0.25 | 1.34 | -0.18 | 0.854 | 0.24 | -0.73 |
|  | Human impact | -0.01 | 0.16 | -0.06 | 0.949 | 0.01 | -0.03 |

Supplementary Table 4B. Results of generalized linear regressions relating detrivorous dung beetle communities as a function of human impact and game mammal abundance in *várzea* forest. All data collected between January 2008 and December 2010 in the Medio Jurua Extractive Reserve and the Uacari Sustainable Development Reserve, Amazonas State, Brazil.

| Model | Coefficient | Est. | SE | *t* | *p-value* | Lower 95% CI | Upper 95% CI |
| --- | --- | --- | --- | --- | --- | --- | --- |
| Community-level biomass | (Intercept) | 0.46 | 0.58 | 0.79 | 0.43 | -0.44 | 1.73 |
|  | Mammal abundance | 0.55 | 1.09 | 0.50 | 0.618 | -0.52 | 3.24 |
|  | Human impact | -0.65 | 0.51 | -1.28 | 0.202 | 0.62 | 1.50 |
| Community-level richness | (Intercept) | 1.68 | 0.37 | 4.57 | 0.000 | -1.62 | 1.09 |
|  | Mammal abundance | 0.39 | 0.67 | 0.59 | 0.559 | -0.38 | 1.99 |
|  | Human impact | -0.26 | 0.30 | -0.88 | 0.382 | 0.25 | 0.87 |
| Dweller species biomass | (Intercept) | -0.05 | 3.67 | -0.01 | 0.99 | 0.05 | 10.86 |
|  | Human impact | -0.20 | 0.90 | -0.23 | 0.529 | 0.19 | 2.66 |
|  | Primate abundance | 4.00 | 6.36 | 0.63 | 0.82 | -3.84 | 18.83 |
| Dweller species richness | (Intercept) | 0.42 | 3.99 | 0.11 | 0.200 | -0.40 | 1.24 |
|  | Human impact | -0.75 | 0.96 | -0.78 | 0.277 | 0.72 | -2.21 |
|  | Primate abundance | 2.80 | 6.83 | 0.41 | 0.529 | -2.69 | 8.3 |
| Roller species biomass | (Intercept) | -3.33 | 2.6 | -1.28 | 0.229 | 3.2 | -9.87 |
|  | Human impact | -0.34 | 0.54 | -0.63 | 0.308 | 0.33 | -1.01 |
|  | Primate abundance | -4.82 | 4.43 | -1.09 | 0.059 | 4.63 | -14.28 |
| Roller species richness | (Intercept) | -1.38 | 1.53 | -0.9 | 0.916 | 1.32 | -4.07 |
|  | Human impact | -0.08 | 0.31 | -0.26 | 0.682 | 0.08 | -0.24 |
|  | Primate abundance | -3.87 | 2.6 | -1.49 | 0.436 | 3.72 | -11.46 |
| Tunneler species biomass | (Intercept) | -4.58 | 3.8 | -1.2 | 0.368 | 4.39 | -13.55 |
|  | Human impact | -1.52 | 0.81 | -1.89 | 0.137 | 1.46 | -4.5 |
|  | Primate abundance | -6.59 | 6.47 | -1.02 | 0.792 | 6.33 | -19.51 |
| Tunneler species richness | (Intercept) | -0.84 | 2.45 | -0.35 | 0.73 | 0.81 | -2.5 |
|  | Human impact | -0.85 | 0.52 | -1.64 | 0.621 | 0.81 | -2.51 |
|  | Primate abundance | -2.05 | 4.15 | -0.5 | 0.100 | 1.97 | -6.07 |
| Biomass species < 0.1g | (Intercept) | -2.22 | 2.79 | -0.79 | 0.427 | 2.13 | -6.56 |
|  | Human impact | -0.48 | 0.60 | -0.81 | 0.547 | 0.46 | -1.43 |
|  | Primate abundance | -2.87 | 4.77 | -0.6 | 0.418 | 2.76 | -8.5 |
| Richness of species < 0.1g | (Intercept) | 0.69 | 1.83 | 0.38 | 0.215 | -0.67 | 2.05 |
|  | Human impact | -0.3 | 0.37 | -0.82 | 0.267 | 0.29 | -0.9 |
|  | Primate abundance* | -0.98 | 3.11 | -0.31 | 0.049 | 0.94 | -2.89 |
| Biomass species ≥ 0.1g | (Intercept) | -3.93 | 3.17 | -1.24 | 0.705 | 3.78 | -11.64 |
|  | Human impact | -1.34 | 0.68 | -1.97 | 0.754 | 1.29 | -3.98 |
|  | Primate abundance | -6.00 | 5.40 | -1.11 | 0.415 | 5.76 | -17.75 |
| Richness of species ≥ 0.1g | (Intercept) | -0.43 | 1.69 | -0.25 | 0.801 | 0.41 | -1.26 |
|  | Human impact | -0.51 | 0.39 | -1.33 | 0.801 | 0.49 | -1.52 |
|  | Primate abundance | -0.73 | 2.9 | -0.25 | 0.184 | 0.7 | -2.17 |
