# Supplementary material for: Human-Induced Trophic Cascades along the Fecal Detritus Pathway
Source: PLoS One. 2013 Oct 16;8(10):e75819. doi: 10.1371/journal.pone.0075819 (PMC3797778; doi:10.1371/journal.pone.0075819)
Supplement: Table S5 — AIC model selection results (A) and generalized linear regression results (B) for AIC top models of dung beetle-mediated secondary seed burial. (DOCX) [file pone.0075819.s006.docx]

Supplementary Table 5A. Results of AIC model selection across alternative models of trophic cascade influence on detritus process rates (probability of secondary seed burial). Seed burial was represented alternatively as a function of the biomass or species richness of either the entire dung beetle community, or using trait-defined community subsets (i.e. defined by body mass or nesting strategy). All data was collected August-October 2009 in 15 *terra firme* forest sites in the Medio Jurua Extractive Reserve and the Uacari Sustainable Development Reserve, Amazonas State, Brazil.

| Process | Metric | Cascade hypothesis | df | AIC | **Δ**AIC | L | W |
| --- | --- | --- | --- | --- | --- | --- | --- |
| **All seeds** | **Biomass** | **Community-level** | **5** | **340.30** | **0.00** | **1.00** | **0.50** |
|  | **Species richness** | **Community-level** | **5** | **340.33** | **0.03** | **0.99** | **0.50** |
| **Large seeds** | **Biomass** | **Body size** | 8 | 152.21 | 0.00 | 1.00 | 0.87 |
|  | Species richness | Body size | 8 | 157.38 | 5.17 | 0.08 | 0.07 |
|  | Biomass | Nesting strategy | 9 | 158.76 | 6.55 | 0.04 | 0.03 |
|  | Species richness | Nesting strategy | 9 | 158.77 | 6.55 | 0.04 | 0.03 |
| **Medium seeds** | **Biomass** | **Body size** | 8 | 231.10 | 0.00 | 1.00 | 0.65 |
|  | Species richness | Nesting strategy | 9 | 233.95 | 2.85 | 0.24 | 0.16 |
|  | Species richness | Body size | 8 | 234.24 | 3.15 | 0.21 | 0.14 |
|  | Biomass | Nesting strategy | 9 | 235.73 | 4.64 | 0.10 | 0.06 |
| **Small seeds** | **Biomass** | **Body size** | 8 | 285.67 | 0.00 | 1.00 | 0.60 |
|  | Species richness | Body size | 8 | 288.16 | 2.49 | 0.29 | 0.17 |
|  | Species richness | Nesting strategy | 9 | 288.66 | 2.99 | 0.22 | 0.14 |
|  | Biomass | Nesting strategy | 9 | 289.54 | 3.87 | 0.15 | 0.09 |

Supplementary Table 5B. Regression results for the top AIC models for fecal detritivore-mediated dispersal of large, medium and small seeds (see STable 5A). All data was collected August-October 2009 in 15 *terra firme* forest sites in the Medio Jurua Extractive Reserve and the Uacari Sustainable Development Reserve, Amazonas State, Brazil.

| Detritus process | Coefficient | Est. | SE | *t* | *p-value* | Lower 95% CI | Upper 95% CI |
| --- | --- | --- | --- | --- | --- | --- | --- |
| All seeds | (Intercept) | -1.85 | 0.30 | -6.13 | 0.000 | -2.45 | -1.26 |
|  | Beetle biomass | 1.17 | 1.30 | 0.90 | 0.369 | -1.38 | 3.71 |
|  | Mammal abundance | -0.56 | 0.65 | -0.86 | 0.391 | -1.83 | 0.72 |
|  | Human impact | -0.27 | 0.56 | -0.48 | 0.628 | -1.37 | 0.83 |
| Large seeds | (Intercept) | -2.76 | 0.94 | -2.93 | 0.003 | -4.61 | -0.92 |
|  | Biomass species < 0.1g* | 6.67 | 2.32 | 2.87 | 0.004 | 2.11 | 11.22 |
|  | Biomass species ≥ 0.1g | -1.59 | 1.70 | -0.94 | 0.349 | -4.93 | 1.74 |
|  | Rodent abundance | -5.74 | 3.71 | -1.55 | 0.122 | -13.02 | 1.53 |
|  | Primate abundance | -2.85 | 2.52 | -1.13 | 0.258 | -7.79 | 2.09 |
|  | Ungulate abundance* | 7.41 | 1.73 | 4.29 | 0.000 | 4.02 | 10.79 |
|  | Human impact | 0.26 | 0.70 | 0.37 | 0.712 | -1.12 | 1.64 |
| Medium seeds | (Intercept) | -1.84 | 0.88 | -2.09 | 0.036 | -3.55 | -0.12 |
|  | Biomass species < 0.1g* | 5.34 | 2.42 | 2.21 | 0.027 | 0.61 | 10.08 |
|  | Biomass species ≥ 0.1g | -1.35 | 1.78 | -0.76 | 0.447 | -4.84 | 2.13 |
|  | Rodent abundance | 1.63 | 3.38 | 0.48 | 0.630 | -5.00 | 8.26 |
|  | Primate abundance | -3.36 | 2.34 | -1.44 | 0.151 | -7.95 | 1.23 |
|  | Ungulate abundance* | 4.94 | 1.72 | 2.86 | 0.004 | 1.56 | 8.32 |
|  | Human impact | 0.02 | 0.77 | 0.03 | 0.976 | -1.48 | 1.53 |
| Small seeds | (Intercept) | -2.67 | 0.69 | -3.87 | 0.000 | -4.02 | -1.32 |
|  | Biomass species < 0.1g* | 3.68 | 1.80 | 2.05 | 0.041 | 0.16 | 7.20 |
|  | Biomass species ≥ 0.1g | 1.46 | 1.35 | 1.08 | 0.281 | -1.19 | 4.11 |
|  | Rodent abundance | 0.20 | 2.64 | 0.08 | 0.940 | -4.98 | 5.38 |
|  | Primate abundance | -4.01 | 1.60 | -2.51 | 0.012 | -7.15 | -0.88 |
|  | Ungulate abundance* | 4.59 | 1.27 | 3.62 | 0.000 | 2.10 | 7.08 |
|  | Human impact | 0.68 | 0.61 | 1.13 | 0.260 | -0.50 | 1.87 |
