# Supplementary material for: Human-Induced Trophic Cascades along the Fecal Detritus Pathway
Source: PLoS One. 2013 Oct 16;8(10):e75819. doi: 10.1371/journal.pone.0075819 (PMC3797778; doi:10.1371/journal.pone.0075819)
Supplement: Table S6 — The Bu basis set of d-separation for three alternative causal path models of the direct and indirect impacts of human activity in terra firme and várzea forests. (DOCX) [file pone.0075819.s007.docx]

Supplementary Table 6. The B_u_ basis set of directional separation (d-sep) claims implied by three causal models of cascade structure described by Figure 2, for *terra firme* (Models A-C) and *várzea* forest (Models D-F). Variable abbreviations: H = human impact, M = game mammals, B = dung beetles, S = seeds. Subscript abbreviations: t = tunneler, r = roller, d = dweller, l = large, m = medium, s = small. *The associated mixed model regression for each d-sep claim used sampling season and transect identity as random effects and appropriate error structures (i.e. Poisson for mammal and beetle models, Binomial for seed models).

| Model | D-sep  independence  claim | Mixed  model  structure* | Variable whose partial regression slope should be zero |
| --- | --- | --- | --- |
| A | (H, B) \| {M} | B ~ M + H | H |
| (k=3) | (H, S) \| {B} | S ~ B + H | H |
|  | (M, S) \| {B+H} | S ~ B + H + M | M |
|  |  |  |  |
| B | (H, B_l_) \| {M_p_+ M_r_+ M_u_} | B_l_ ~ H + M_p_ + M_r_ + M_u_ | H |
| (k=21) | (H, B_s_) \| {M_p_+ M_r_+ M_u_} | B_s_ ~ H + M_p_ + M_r_ + M_u_ | H |
|  | (H, S_l_) \| {B_l_ + B_s_} | S_l_ ~ H + B_l_ + B_s_ | H |
|  | (H, S_m_) \| {B_l_ + B_s_} | S_m_ ~ H + B_l_ + B_s_ | H |
|  | (H, S_s_) \| {B_l_ + B_s_} | S_s_ ~ H + B_l_ + B_s_ | H |
|  | (M_p_, S_l_) \| {B_l_ + B_s_ + H} | S_l_ ~ M_p_ + B_l_ + B_s_ + H | M_p_ |
|  | (M_p_, S_m_) \| {B_l_ + B_s_ + H} | S_m_ ~ M_p_ + B_l_ + B_s_ + H | M_p_ |
|  | (M_p_, S_s_) \| {B_l_ + B_s_ + H} | Ss ~ M_p_ + B_l_ + B_s_ + H | M_p_ |
|  | (M_r_, S_l_) \| {B_l_ + B_s_ + H} | S_l_ ~ M_r_ + B_l_ + B_s_ + H | M_r_ |
|  | (M_r_, S_m_) \| {B_l_ + B_s_ + H} | S_m_ ~ M_r_ + B_l_ + B_s_ + H | M_r_ |
|  | (M_r_, S_s_) \| {B_l_ + B_s_ + H} | S_s_ ~ M_r_ + B_l_ + B_s_ + H | M_r_ |
|  | (M_u_, S_l_) \| {B_l_ + B_s_ + H} | S_l_ ~ M_u_ + B_l_ + B_s_ + H | M_u_ |
|  | (M_u_, S_m_) \| {B_l_ + B_s_ + H} | S_m_ ~ M_u_ + B_l_ + B_s_ + H | M_u_ |
|  | (M_u_, S_s_) \| {B_l_ + B_s_ + H} | S_s_ ~ M_u_ + B_l_ + B_s_ + H | M_u_ |
|  | (M_p_, M_r_) \| {H} | M_r_ ~ M_p_ + H | M_p_ |
|  | (M_r_, M_u_) \| {H} | M_u_ ~ M_r_ + H | M_r_ |
|  | (M_p_, M_u_) \| {H} | M_u_ ~ M_p_ + H | M_p_ |
|  | (B_l_, B_s_) \| {B_l_ + B_s_} | B_s_ ~ B_l_ + H + M_p_ + M_r_ + M_u_ | B_l_ |
|  | (S_l_, S_m_) \| {B_l_ + B_s_} | S_m_ ~ S_l_ + B_l_ + B_s_ | S_l_ |
|  | (S_l_, S_s_) \| {B_l_ + B_s_} | S_s_ ~ S_l_ + B_l_ + B_s_ | S_l_ |
|  | (S_m_, S_s_) \| {B_l_ + B_s_} | S_s_ ~ S_l_ + B_l_ + B_s_ | S_m_ |
|  |  |  |  |
| C | (H, B_t_) \| {M_p_+ M_r_+ M_u_} | B_t_ ~ H + M_p_ + M_r_ + M_u_ | H |
| (k=24) | (H, B_r_) \| {M_p_+ M_r_+ M_u_} | B_r_ ~ H + M_p_ + M_r_ + M_u_ | H |
|  | (H, B_d_) \| {M_p_+ M_r_+ M_u_} | B_d_ ~ H + M_p_ + M_r_ + M_u_ | H |
|  | (H, S_l_) \| {B_t_ + B_r_ + Bd} | S_l_ ~ H + B_t_ + B_r_ + B_d_ | H |
|  | (H, S_m_) \| {B_t_ + B_r_ + Bd} | S_l_ ~ H + B_t_ + B_r_+ B_d_ | H |
|  | (H, S_s_) \| {B_t_ + B_r_ + Bd} | S_l_ ~ H + B_t_ + B_r_+ B_d_ | H |
|  | (M_p_, S_l_) \| {B_t_ + B_r_ + B_d_ + H} | S_l_ ~ M_p_ + B_t_ + B_r_ + B_d_ + H | M_p_ |
|  | (M_p_, M_l_) \| {B_t_ + B_r_ + B_d_ + H} | S_l_ ~ M_p_ + B_t_ + B_r_ + B_d_ + H | M_p_ |
|  | (M_p_, S_s_) \| {B_t_ + B_r_ + B_d_ + H} | S_l_ ~ M_p_ + B_t_ + B_r_ + B_d_ + H | M_p_ |
|  | (M_r_, S_l_) \| {B_t_ + B_r_ + B_d_ + H} | S_l_ ~ M_r_ + B_t_ + B_r_ + B_d_ + H | M_r_ |
|  | (M_r_, S_m_) \| {B_t_ + B_r_ + B_d_ + H} | S_m_ ~ M_r_ + B_t_ + B_r_ + B_d_ + H | M_r_ |
|  | (M_r_, S_s_) \| {B_t_ + B_r_ + B_d_ + H} | S_s_ ~ M_r_ + B_t_ + B_r_ + B_d_ + H | M_r_ |
|  | (M_u_, S_l_) \| {Bt + B_r_ + B_d_ + H} | S_l_ ~ M_u_ + B_t_ + B_r_ + B_d_ + H | M_u_ |
|  | (M_u_, Sm) \| {Bt + B_r_ + B_d_ + H} | S_m_ ~ M_u_ + B_t_ + B_r_ + B_d_ + H | M_u_ |
|  | (M_u_, S_s_) \| {Bt + B_r_ + B_d_ + H} | S_s_ ~ M_u_ + B_t_ + B_r_ + B_d_ + H | M_u_ |
|  | (M_p_, M_r_) \| {H} | M_r_ ~ P_u_ + H | M_p_ |
|  | (M_r_, M_u_) \| {H} | M_u_ ~ M_r_ + H | M_r_ |
|  | (M_p_, M_u_) \| {H} | M_u_ ~ P_u_ + H | M_p_ |
|  | (B_t_, B_r_) \| {M_p_ + M_r_ + M_u_} | B_r_ ~ B_t_ + M_p_ + M_r_ + M_u_ | B_t_ |
|  | (B_r_, B_d_) \| {M_p_ + M_r_ + M_u_} | B_d_ ~ B_r_ + M_p_ + M_r_ + M_u_ | B_r_ |
|  | (B_t_, B_d_) \| {M_p_ + M_r_ + M_u_} | B_d_ ~ Bt + M_p_ + M_r_ + M_u_ | B_t_ |
|  | (S_l_, S_m_) \| {B_t_ + B_r_ + B_d_} | S_m_ ~ S_l_ + B_t_ + B_r_ + B_d_ | S_l_ |
|  | (S_l_, S_s_) \| {B_t_ + B_r_ + B_d_} | S_s_ ~ S_l_ + B_t_ + B_r_ + B_d_ | S_l_ |
|  | (S_m_, S_s_) \| {B_t_ + B_r_ + B_d_} | S_s_ ~ S_m_ + B_t_ + B_r_ + B_d_ | S_m_ |
|  |  |  |  |
| D | (H, S) \| {B} | B ~ M_p_ + H | H |
| (k=1) |  |  |  |
|  |  |  |  |
| E | (H, B_l_) \| {M_p_} | B_l_ ~ M_p_ | H |
| (k=3) | (H, B_s_) \| {M_p_} | Bs ~ M_p_ | H |
|  | (B_l_, B_s_) \| {M_p_} | B_l_ ~ B_s_ + M_p_ | B_l_ |
|  |  |  |  |
| F | (H, B_t_) \| {M_p_} | B_t_ ~ H + M_p_ | H |
| (k=6) | (H, B_r_) \| {M_p_} | B_r_ ~ H + M_p_ | H |
|  | (H, B_d_) \| {M_p_} | B_d_ ~ H + M_p_ | H |
|  | (B_t_, B_r_) \| {M_p_} | B_r_ ~ B_t_ + M_p_ | B_t_ |
|  | (B_r_, B_d_) \| {M_p_} | B_d_ ~ B_r_ + M_p_ | B_r_ |
|  | (B_t_, B_d_) \| {M_p_} | B_d_ ~ B_t_ + M_p_ | B_t_ |
